# A Profile of Occupational Therapists Working in School-Based Practice in Australian Primary Schools

**DOI:** 10.1155/2024/2077870

**Published:** 2024-04-26

**Authors:** Jill Jeremy, Ilektra Spandagou, Joanne Hinitt

**Affiliations:** ^1^Sydney School of Education & Social Work, Faculty of Arts & Social Science, The University of Sydney, Camperdown, Australia; ^2^Sydney School of Health Sciences, Faculty of Medicine and Health, The University of Sydney, Camperdown, Australia

## Abstract

Inclusive education has increased the demand for school-based occupational therapy services and has reconceptualised the practice in mainstream schools. Therapists are now expected to work collaboratively with teachers within tiered intervention models to support access and participation of all students, including those with disabilities, within the natural classroom context. School-based occupational therapy has become a specialised area of practice, as therapists work within educational, rather than health, systems and processes. While the growth in demand and expanded scope of practice is positive for the profession, predicted workforce shortages and the necessity for specialised and enhanced practice present significant challenges. The ability of the profession to fully support the demands of an inclusive education system remains unclear. As accurate, up-to-date information on the school-based therapy workforce is the foundation for planning future personnel needs, knowledge of the current state of the workforce is critical. There is a paucity of national data regarding this growing area of practice. The aim of this study is to describe a current profile of school-based occupational therapists to better understand the workforce, practice patterns, and the funding landscape in Australia. A convenient and purposive sample of 108 Australian paediatric occupational therapists working in mainstream primary schools in New South Wales, Queensland, and Victoria was surveyed in this quantitative study, which was analysed using descriptive statistics. Results provide some insights into the workforce and practice of school-based therapy in Australia offering preliminary data for future planning in this important and growing area of paediatric practice. While specific to the local context, results invite cross-national and global comparison to reveal universal trends and localised nuances across diverse settings.

## 1. Introduction

The field of occupational therapy currently exhibits optimism regarding job prospects for its personnel as employment opportunities are increasing in various areas of practice [1]. One particular area of growth is school-based practice, largely fuelled by the increasing emphasis on inclusive education and a corresponding rise in the number of children with disabilities being schooled in the mainstream [2]. As the World Federation of Occupational Therapists [3] promotes school-based occupational therapy practice as a means of supporting the rights of all children to an inclusive education, demand has increased for therapists to practice in mainstream settings, fundamentally reshaping their role within the education system. Occupational therapists are now expected to work collaboratively with teachers within tiered intervention models to support *all* students in their classroom environment.

While increased demand and expanded scope present opportunities, impending workforce shortages and the need for advanced practice capabilities present challenges. It remains unknown whether the profession will have the capacity and capability to meet the needs of an evolving inclusive education system. Workforce data provides a foundation for examining current school-based service provision and the occupational therapy workforce in this area. Up-to-date workforce data allows the mapping of current service provision to identify underserved areas, evaluate program effectiveness, and advocate for increased resources. Data may also be used to project future needs, develop targeted recruitment strategies, design effective training programs, and support professional development. Comprehensive workforce data is therefore necessary to ensure the accessibility, effectiveness, and sustainability of school-based OT services, benefiting students, teachers, and the profession as a whole.

There is a paucity of data regarding the provision of school-based occupational therapy services and the nature of occupational therapists' work within this field [4, 5]. This study is aimed at addressing this lack by describing the current state of the school-based occupational therapy workforce in Australia. By understanding the workforce, practice patterns, and the funding landscape, critical gaps may be identified, and a plan created to ensure a sustainable workforce equipped to address the needs of inclusive systems of education. The study illustrates the landscape of school-based occupational therapy in Australia using a small purposive sample of occupational therapists working in mainstream primary schools in three Australian states. The resulting profile includes workforce information, such as demographics, employment, and workplace; practice characteristics, such as the client base and models of service delivery; and funding information, including sources and sustainability.

### 1.1. Paediatric and School-Based Occupational Therapy Workforce

Workforce is the single most valuable resource of any organisation, and workforce planning is an essential component of a system's ability to train, recruit, and retain a “fit for purpose” workforce to effectively meet existing and future needs [6]. Previous studies examining the international occupational therapy workforce predicted a workforce shortage [7], confirmed by current reports of a global scarcity of occupational therapists ([8–10]. These workforce shortages place the occupational therapy workforce under huge pressure and challenge therapists' capacity to meet the needs of the systems that they work within, including education. To fulfill their professional role in the education system, occupational therapists need to be in adequate supply, and sufficiently qualified and experienced, to deliver services using best-practice models.

Paediatric practice is a common area of employment within the international occupational therapy profession [11, 12]. In the USA, almost 30% of occupational therapists work in early intervention and schools [13], and school-based practice is a growing area of employment globally [1]. In Australia, the increasing demand for paediatric occupational therapists is likely a direct result of the introduction of the National Disability Insurance Scheme (NDIS) [14]. This scheme, initially trialled in 2013 and subsequently rolled out nationally reaching full coverage in 2020, shifted the provision and funding of disability services from block funding of government and nongovernment services to individualised budgets, consumer choice, and fee for service models, increasing the demand for paediatric practitioners to support funded children [15]. The increase in therapists practising under this scheme may explain the growth in paediatrics as a principal scope of practice, from 18.4% of the occupational therapy workforce in 2015 to 20.3% in 2019. Although anecdotal reports suggest a corresponding increase in paediatric therapists working in schools, there is little available data on this population and the services that they provide. The total occupational therapist workforce in Australia increased by 31.1% over five years, from 18,304 in 2015/16 to 23,997 in 2019/20. Despite an average annual growth of 7%, a predicted shortage of occupational therapists remains as demand is predicted to exceed supply. Current directions in health, aged and disability care, changing population demographics, and increasing service awareness and accessibility suggest that workforce challenges will continue for the foreseeable future [8].

Recent data on the occupational therapy workforce reports a 90.6% female and 9.4% gender division. The average age of practitioners was 37.3 years, with 42% of the workforce aged under 35 years and 9.3% aged 55 years and older [8]. The proportion of occupational therapists in each state varies, with 28.4% of registered occupational therapists practising in New South Wales, 26.2% in Victoria, and 20.7% in Queensland. The mean number of years in practice was 10.9 years. More than half (55.5%) of therapists were employed in the private sector, with 20% reporting their principal place of practice as paediatrics, and 5% reporting their principal work setting as education [16]. Workforce profiling of occupational therapists provides insight into the composition of the sector's workforce, highlighting the demographics, skills, and experience of practitioners within the system. An understanding of the current workforce is essential to support benchmarking, workforce planning, and employment policy development and to monitor the impact of workforce reforms.

Paediatrics is a common area of practice for occupational therapists, and demand for occupational therapy in children's services is increasing [10]. Within paediatrics, there is a growing recognition of the importance of occupational therapy in schools, and school-based occupational therapy is considered the best practice for supporting inclusion in educational settings [17]. Described as occupation-focussed, educationally relevant, and contextual [3], services within this model are generally organised using an interrelated three-tiered framework of intervention that addresses needs at all levels of the education system [18]. Tier 1 interventions provide universal service through universal design for learning and bidirectional capacity building of teachers and therapists. Tier 2 interventions provide targeted service through differentiated instruction to children at risk of, or already experiencing, difficulties. Tier 3 interventions provide tailored service through environmental or learning accommodations provided to individual children who have complex needs requiring a personalised approach [18]. Although the literature supports using a blend of tiered interventions to effectively meet the diverse needs of students [19, 20], countries' different health and educational systems, and different employment practices for therapists, impact therapists' scope to deliver intervention using these tiers [4].

Differences in health and education legislation internationally, and in how legislation is enacted in policy, have resulted in different systems of occupational therapy service provision to students in school [20–22]. Unlike the USA, where therapy is mandated as a “related service” for students with “special education needs,” there is no federal legislation in Australia that legally or structurally embeds occupational therapists in the education system; thus, the presence of occupational therapists in Australian schools is varied. Although each Australian state and territory has policies that support the provision of occupational therapy in schools, differences in employment, funding, and service delivery between the jurisdictions have resulted in incredible complexity of service provision and delivery practices within and between states and territories [22]. Depending upon the jurisdiction, therapists may be directly employed by an education department, employed or contracted by a school, or employed by external providers to provide individual services to students in schools. There is no current Australian workforce data that profiles the nature of occupational therapists' work in mainstream schools. It is not known how many occupational therapists provide services to students or to schools, nor are the demographic profile, experience, location, context, funding, or models of service provision understood. Thus, there is a need to adequately describe the presence, employment, and funding of occupational therapists in school-based practice in Australia.

Research on occupational therapists working in Australian education systems is scarce, and the majority is dated. Only four studies were found that described a profile of practice. Two articles, from 30 years ago, describe the same study of paediatric therapy service provision in the state of Queensland [23, 24]; one article from almost 20 years ago describes a profile of Australian paediatric occupational therapy practice [25]; one article describes the paediatric occupational therapy workforce in schools in the state of Victoria [26], and the most recent study describes the role of occupational therapy in government-funded primary schools in Australia [22].

In the study of paediatric therapy service provision in the state of Queensland [23, 24], 146 occupational therapists with a paediatric caseload, identified through the Queensland Association of Occupational Therapists database, were invited to complete a survey soliciting workplace activities including service delivery information. Of the 87 respondents, the majority (74%) worked full time, with 63% working in hospital or private practice and 11.5% in educational facilities. Educational facilities were ranked as the third most frequently used venue for service provision. School programs composed 10% of all service provision, and 98% of respondents had contact with primary-aged children, with 57% working with this client group most frequently. Respondents most commonly worked with a caseload of children with a variety of disabilities. Individual intervention (tier 3) was the most common method of service provision followed by group programs (tier 2). No therapists in this study used tier 1 service as their main means of intervention. Although the workforce profile detailed in this study is aged and not specific to school-based practice, it does provide some information on the profile of paediatric occupational therapy practice in this state.

The national study [25] used a purposive sample of 600 paediatric occupational therapists, identified through Occupational Therapy Australia's database, to recruit 330 respondents to a survey questionnaire. This study described a profile of Australian paediatric occupational therapy practice as part of a larger international study comparing paediatric practice in Australia, New Zealand, and Canada. The majority of respondents were from Victoria (33%), followed by Queensland (27%), and New South Wales (13%). Most respondents were female (97%), aged between 30 and 39 years (38%), had worked as an occupational therapist for 14 years and in paediatrics for 11 years, and held a bachelor's degree (82%). Of the respondents, 66% worked full time (20-40 hours), 15% in private practice, and 13% employed by a school board or educational system. Caseloads were typically community-based (77%) with primary school-aged children as the primary recipients of service (38%). One of the main findings of this study, when compared to Rodger et al. [23], was the shift from hospital to community paediatric practice. This study provides some demographic, employment, and service provision data; however, the focus was on theories, assessments, and interventions.

Latterly, a study conducted by Occupational Therapy Australia [26] in the state of Victoria is aimed at better understanding the existing workforce of occupational therapists working in schools in this state. Using purposive convenience and snowball sampling of the Victorian paediatric occupational therapy community, 184 respondents completed an online survey questionnaire. Of the respondents, 51% worked in state primary schools, 25% in special education schools, 12% in Catholic education schools, and 6% in independent schools. Most respondents (64%) worked with between 1 and 3 schools; however, 36% worked between 5 and 30 schools, with 1% working with more than 30 schools. Respondents worked a variety of hours in school-based practice; 16% worked full time (more than 30 hours) while 33% worked only 1 hour per week in schools. Of the therapy services provided at school, the most frequent provision was direct 1 : 1 therapy with a specific child (tier 3). Other services provided were direct small group (tier 2) and consultation with teachers and/or school support staff (tier 1). The most common method of funding services was NDIS funding, with 35% of respondents paid using this source. For those working in special education schools, Victorian Department of Education funding was most frequently used to pay for services. Fourteen percent (14%) of respondents reported receiving payment using private funding methods, such as parent payments or private health insurance. A variety of other funding measures were reported but were used very infrequently. With regards to employment, 45% of respondents were sessional or casual. For those with school contracts, 20% had an ongoing contract, 10% had an annual contract, and 1% had a termly contract. While the focus of the study was on school employment and funding, it also provides a partial work profile of occupational therapists working in this area. Although there were acknowledged limitations of this study, it confirmed that the current school-based occupational therapy workforce in Victoria is very diverse, is supported through a variety of funding sources, and provides an assortment of services using a range of models.

Most recently, a small qualitative study [22] of 12 occupational therapy representatives from each of the eight Australian states and territories described the role of occupational therapy in state primary schools. This study reports that all Australian states and territories have some form of school-based occupational therapy service provision but that it is diverse and difficult to describe due to the different employment models used. Therapists may be directly employed by state or territories' education departments and work between several different government schools. Queensland's Department of Education has a thirty-year history of directly employing occupational therapists in this manner, and other states, such as Victoria and South Australia, appear to be adopting this model too. In the jurisdictions where education departments do not employ occupational therapists directly, individual schools may choose to engage a therapist, who may work at various schools or be employed full time at one school. Occupational therapists practising within this model are usually in private practice or employees of an organisation contracted to provide services to schools. This model occurs to varying degrees in most states and territories. External provision of therapy services to individual students in schools, whether services are provided through an organisation or through sole traders in private practice, is common in all states and territories, though particularly prevalent in New South Wales. Provision of services in this manner is at the discretion of the individual school principal, who makes decisions regarding the delivery of services at schools in light of practical, legal, and educational considerations, and thus may choose to limit or prevent delivery of externally funded services. A myriad of service models was utilised by occupational therapists in schools, ranging from direct 1 : 1 pull-out (tier 3) through to whole school consultation (tier 1). This study provides an overview of the presence, employment, funding, and service models of occupational therapists in education.

Although these four studies provide background data on occupational therapists working in schools in Australia, the information is outdated, piecemeal, and insufficient to establish a profile of current practice or to offer useful workforce planning information. Without this information, it is difficult to understand the unique work and context of school-based practice, or to plan for future workforce demands in this area. To meet the future needs of the education system, current knowledge of practitioners' presence and employment in mainstream schools is essential, as inclusive education requires an adequate supply of sufficiently qualified and experienced school-based occupational therapists to deliver services using best-practice models. Current and detailed occupational therapy workforce data is required to ensure the capacity and capability of occupational therapists to deliver national and international strategic priorities regarding inclusive education, now and in the future [27].

### 1.2. Purpose

The purpose of this study was to describe a profile of a sample of occupational therapists working in schools in three Australian states. The research questions were as follows:

- What are the demographic and employment characteristics of occupational therapists providing school-based services?
- What therapy services do occupational therapists provide in schools?
- Who are the clients receiving these therapy services?
- How are the therapy services funded?

## 2. Methods

The University of Sydney Human Research Ethics Committee provided ethical approval (Project Number 2021/278) prior to the commencement of recruitment and survey distribution.

### 2.1. Study Design

This study forms part of a larger study investigating collaborative practice between occupational therapists and teachers in Australian primary schools. The study employed a quantitative, cross-sectional study design using an online survey questionnaire, allowing respondents to participate easily and cheaply, as well as offering a rapid data collection turnaround time.

### 2.2. Sample

Nonprobability techniques of convenience sampling, self-selection, and passive snowballing were employed. Participants were recruited by several methods including through social and professional networks, by advertising on Occupational Therapy Australia's (OTA) website and their fortnightly email to members, and target emailing therapists who identified as paediatric providers on the OTA provider website. Study participants were occupational therapists currently working in mainstream primary schools or providing therapy services to individuals in schools, who were qualified and registered to practice in one of the three states with the Occupational Therapy Board of Australia and who voluntarily consented to the study. The three states, New South Wales, Queensland, and Victoria, were chosen as they are the most populous states, and between them, they employ 75% of the occupational therapists in Australia [16]. As models of school-based therapy provision vary between these states [22], they were judged to be representative of the diversity of service provision. Workforce data suggests that approximately 3,000 occupational therapists work in paediatric practice within these states with approximately 740 working in an educational setting [16]. As the actual number of therapists working in mainstream primary schools is unknown, the sample size was not predetermined, and participants were continuously sought until survey closure.

### 2.3. Data Collection

Data collection occurred over a 17-month period. This extended time period was necessary as COVID-19 impacted occupational therapists' work in schools, as well as their time and availability to participate in the research.

The anonymous online survey questionnaire was hosted by REDCap, a secure web application for building and managing online surveys and databases. Participants accessed the survey via a generic link and, prior to survey commencement, were asked to read the participant information sheet and consent form, confirm their eligibility, and provide consent via submission of the completed survey.

### 2.4. Survey Instrument

An anonymous web-based survey questionnaire was developed to answer the questions of the larger study. This contained 70 closed questions over 3 sections: demographics and background data (section 1), Teacher-Therapist Collaboration Index (section 2), and influences on collaboration (section 3), plus an additional comment section. The questionnaire items from section 1, which included questions on demographics, background, employment, service delivery, and funding, were used to answer the research questions for this study.

The steps of survey construction were used to substantiate the validity and reliability of the survey questionnaire [28]. Prior to survey development, a review of the paediatric and school-based occupational therapy literature was undertaken, and existing surveys were reviewed. School-based therapists were consulted to provide feedback, and based on their feedback, changes were made to refine and clarify questions and structure. Finally, to establish validity [29], a pilot test was administered to a sample of five occupational therapists with experience working in schools. This approach, incorporating expert evaluations, participant feedback, and empirical testing, established a foundation for the content validity of the survey instrument.

### 2.5. Data Analysis

This study reports on the data collected in the demographic and background section of the survey. Quantitative descriptive statistics describing and summarising the dataset were analysed using IBM SPSS statistics (version 28) and are presented below.

## 3. Results

As the population of school-based occupational therapists in Australia is unknown, as is the total number of individuals who received the survey link, a response rate could not be determined. One hundred and eight occupational therapists (*n* = 108) participated in the study. As not all participants answered every question, missing data occurred, and results are presented for the number of responses received for each question.

### 3.1. Demographic and Employment Characteristics of School-Based Therapists

Table 1 presents the demographic characteristics of respondents by state. Most respondents were from New South Wales (*n* = 57), followed by Victoria (*n* = 30), then Queensland (*n* = 18). The vast majority of occupational therapists who responded were female (94%), held a bachelor's degree (71%), were between 26 and 35 years of age (42.9%), and had been in the profession under nine years (46.7%). There was variation between respondents' characteristics. The proportion of male therapists was higher in Queensland (11%) than in Victoria (7%) and New South Wales (4%). The proportion of therapists in the 26- to 35-year and 36- to 45-year age groups also differed between the states. Queensland reported 33.3% and 38.9%, respectively, while New South Wales reported 43.9% and 26.3%, and Victoria reported 46.7% and 23.3%. Victorian respondents had a higher proportion of therapists with a master's degree (33.3%) compared to New South Wales (28.1%) and Queensland (22.2%). With regards to experience, almost half of respondents (46.7%) had less than nine years of experience, while 9.6% had over 30 years of experience. The percentage of therapists with nine and less years of experience was higher in Queensland (61.1%) than in New South Wales (43.9%) and Victoria (43.3%).

Table 2 presents the employment characteristics of respondents by state. Slightly more than half of the respondents (55%) worked full time. Of those working part time (45%), the number of hours worked each week ranged from 10 to 37 hours. There was little difference between states with regard to the number of hours worked. The vast majority of occupational therapists (64%) worked in private practice, although 16% indicated that they were employed by the Department of Education, and 16% worked for an agency or not-for-profit organisation. Employment sector varied considerably between states, with 79% of respondents in New South Wales (*n* = 37) working in private practice, 58% in private practice in Victoria (*n* = 14), while only 33% of Queensland respondents (*n* = 6) reported working in this sector. Conversely, 50% of Queensland respondents were employed through the education department of their state government, while this figure was 17% in Victoria, and only 2% in New South Wales.

### 3.2. Services and Service Delivery in School-Based Practice

Table 3 presents the services provided by school-based occupational therapists and the models of service delivery that they use. Therapists provided a range of services, using a variety of models generally consistent with the 3-tiered framework. The vast majority of therapists (73.1%) provided tier 3 services, while only 24.1% provided tier 2 services. The proportion of therapists providing tier 1 services differed depending upon whether services were provided to a whole class or a whole school, with more therapists in Queensland and Victoria providing these services than in New South Wales. Other services provided included consulting with teachers about individual students (66%), or providing indirect therapy (29%), where a program of intervention is developed by a therapist but implemented by education staff. More Queensland and Victorian therapists provided indirect services (39% and 37%, respectively) than therapists in New South Wales (21%).

Sixty-one percent (61%) of therapists most frequently provided services using tier 3 direct one to one therapy with individual students. Direct small group and class consultation services were least frequently provided, with only 3% of therapists noting this as a service frequently provided. Individual consultation was more frequently practiced in Queensland (44%) than in New South Wales or Victoria (21% and 26%, respectively). Although tier 3 service provision was most frequently provided using a pull-out model located within a room in the school, the percentage of in-class tier 3 service provision was higher in Queensland (39%) and Victoria (36%) than in New South Wales (28%). Tier 2 small group services were more often located in the natural environment of the classroom, but this varied between states with those in Queensland providing in-class service more often than the other two states. Services were most often provided during class time (85%); however, 13% of therapists provided in-school services prior to or after the school day. Service provision during recess or lunch was rare.

With regards to therapists' contributions to individual student education plans, few therapists (2%) contributed to all plans for students for whom they provided services. Nonetheless, almost half (49%) contributed to some student plans. Fourteen percent (14%) of therapists were unaware of which of their students had education plans.

Therapists worked with students across all primary grades, from the first to the last years of schooling. Service provision was most common in the early years, from the first to the third year of schooling, with 48% of therapists providing services to students in their first year of school and 20% providing services to those in their second year. Only 4% of therapists provided services to students in the upper years of primary school.

### 3.3. Clients of School-Based Occupational Therapists

Table 4 reports the clients that received services from the occupational therapists. Clients varied from individual students and individual classroom teachers through to small groups of students, and all school staff, depending on the service delivery model employed. Therapists worked with both teachers and students. The mean number of teachers that therapists were working with was 14. This ranged from 13 teachers in New South Wales to 17 in Victoria. The actual number of teachers working with therapists ranged from 2 to 40. The mean number of students on a therapists' caseload was 22, and, although this did not vary much between states, the range was 1 to 67 students, with 8% of therapists holding a caseload of 40 students.

Of the students on a therapists' caseload, a mean of 18 students had a diagnosed disability. This figure was slightly higher in New South Wales and Queensland, with 21 students in both states, than in Victoria (18 students). The mean number of students receiving adjustments per the NCCD was 11, with the number lower in New South Wales (9 students) and higher in Victoria (13 students) and Queensland (14 students). Of those students on a therapist's caseload, 61% were receiving school-based services, though this figure was lower in Victoria (50%) and New South Wales (31%) and higher in Queensland (72%).

### 3.4. Funding of School-Based Therapy Services

Table 5 details the sources of funding of therapy services. Therapy services were funded via a variety of means. The most common funding source was individual funding through the NDIS (76%), with 85% of therapists in New South Wales reporting that their services were funded this way “always” or “frequently.” In Queensland, NDIS funding was used less frequently, with 47% of therapists reporting that services were funded this way. Private funding accounted for almost one-fifth of services (19%) and was more commonly used in New South Wales (27%) than in Queensland (24%) and Victoria (19%). Government funding, via departments of education, was more commonly used in Queensland with 56% of therapists reporting that this funding source was used “always” or “frequently,” compared to only 7% of therapists in New South Wales. Similarly, Catholic education or independent sector funding and individual school funding were more commonly used to fund services in Queensland than in the other two states.

## 4. Discussion

The results of this small, preliminary study concur with Pozorski et al.'s [22] assertion that, although all states and territories have some form of school-based occupational therapy service provision, there is huge variation in presence, employment, service delivery, and funding of occupational therapy in the Australian education system. These differences are important considerations when creating national policy and for equitable access to, and receipt of, services.

The demographic profile of this sample's respondents is similar to previous studies with the profession remaining predominantly early middle-aged, female, and holding a bachelor's degree. Occupational therapy remains a gender-segregated profession with little change in gender balance over time. Gender parity in employment is important, both as a tool to promote gender equality and to ensure that both genders can access the same professional opportunities [30]. As a potential strategy to resolve the workforce shortage, future occupational therapy training, recruitment, and employment policies could address both the gender imbalance, to encourage more males into the profession, as well as incentivising the master's degree, to encourage graduates of other disciplines to retrain as an occupational therapist.

Study respondents reported a higher number of years of experience than the national average, but less than Rodger et al.'s study [25] of paediatric practitioners. It may be that early career therapists lack the required unique knowledge and skills to work in school-based practice and that it is therefore more suited to experienced therapists. The unique nature of the knowledge and skills required to work in school-based practice means that therapists feel poorly prepared for this practice area with entry-level education alone [14]; thus, the onus is on tertiary occupational therapy education programs to ensure that graduates have the requisite skills for this complex area of practice upon graduation, and the profession may need to provide better workforce supervision and support for early career professionals entering this practice specialty [14]. The respondents from Queensland who had the least experience and were more commonly employed by the Department of Education may suggest that this model of employment provides the necessary supports and supervisions for less experienced therapists to feel confident to practice in this area, although the numbers are too small to generalise. At the other end of the experience spectrum, one-tenth of respondents had more than 30 years of experience, with 7.6% of respondents aged over 56 years of age. Although recent data suggests a replacement rate of 5.1, that is, five therapists entering the workforce for every one that leaves [16], the predicted future demand for occupational therapy services raises questions related to the professions' preparedness for an ageing workforce, its ability to replace retiring professionals in the coming decades, and the concurrent caring responsibilities of this demographic.

It is difficult to compare the working hours of occupational therapists across studies as the interpretation of full time differed; however, slightly more than half (55%) of study respondents worked more than 38 hours per week, with 45% of therapists currently working part time. Encouraging the part-time workforce into full-time employment may also be a solution to meet the increasing demand for therapists.

Funding has a major effect on how services are provided, and funding bodies operate within their own directives regarding service delivery [31]. Funding in this study varied between states and came from a variety of sources, though most commonly through the NDIS. The NDIS is designed to provide supports and services that are not funded by other government services, and as schools are obliged to provide reasonable adjustments to ensure that students with disabilities can access and participate in their education, the services offered by an occupational therapist via NDIS funding should not be used for educational purposes. Guidelines exist to delineate what the NDIS can and cannot fund in schools; however, as there are some areas of overlap, the interpretation of these can be confusing [32]. Understanding the limitations of the scheme within the education sphere is an important factor in school-based occupational therapy service provision, as stakeholders must identify when NDIS-funded services can and cannot be engaged to support students [33].

The differences between the states regarding funding of services via Departments of Education raise questions of equity in the receipt of services in school between the states. Education department funding was a common source of funding of therapy services in Queensland, and an incredibly uncommon source of funding in New South Wales, reflecting the different ways that services are funded and provided in these two states. Private funding was commonly used in all three states. Although little literature exists on the extent of service provision funded via private means, access to therapy services is impacted if provision depends on an individual's level of private health insurance coverage, or the families' ability to pay out-of-pocket expenses, which also has implications for equity of service provision across the states.

That service provision varied between states is unsurprising given that different employment and funding models impact a therapist's ability to use some service delivery models [18]. A therapist in private practice, contracted to provide individual services to a student through NDIS funding, is prohibited from using tier 1 universal service provision models, such as whole class or school consultation. Consistent with the literature, tier 3 one-on-one pull-out remained the most common method of service delivery in this study [34], and the most common clients were students in the early years of schooling. So, few students in the upper years received therapy services, which may be a result of the profession's focus on early intervention; however, it raises the question as to whether the older client population is underserved. The provision of services to students in upper primary and in secondary education is an area requiring further investigation.

Those occupational therapists contributed to only half of the individualised education plans of students that they were seeing suggesting that occupational and educational goals are not in alignment. It may be that the way that therapy services are funded and provided prevents therapists from contributing to educational goals. This is also an area which needs further examination given the importance of a holistic approach to including students with disabilities.

Although a blend of delivery approaches remains essential to effectively meet student needs [20], inclusion demands the use of models that are contextually based in the natural setting of the classroom [35] and which provide multitiered systems of support with a focus on collaboration [36, 37]. That between one-quarter and one-fifth of therapists also provided tier 1 whole class, or whole school consultation is promising. The expansion of inclusive education warrants a change in service delivery models, from costly and time-consuming individual-based interventions towards capacity-building models [38] requiring therapists to identify activities that they are providing to schools beyond traditional caseloads.

That caseload models, where therapists have a number of students that they see, were typical of respondents in this survey and are not surprising given the models that the therapists work under. However, these traditional “counting” approaches do not recognise the complexity of the occupational therapy role in current best practice, nor do these clinical pull-out models adequately support inclusion or the generalisation of skills to the natural environment of the classroom [39]. Caseload models also prevent the recognition of the potential of occupational therapy to contribute to access and participation goals for all students, which are inherent in inclusive environments. Thus, to meet the needs of students, teachers, and schools, therapists may need support to move from a caseload to a workload approach [40]. Such an approach encompasses a multitiered system and recognises all work activities performed that benefit students, directly and indirectly, including activities directed towards groups of students, whole classrooms, or school-wide populations [41]. Workload approaches require therapists to redesign their work patterns to serve students in the classroom context to support performance needs, as well as creating time in their day for collaborative teamwork and data collection. This will necessitate both a paradigm shift and a system change in funding and employment structures so that therapists are supported to adjust their model of service delivery to better meet the needs of the education system.

The purpose of this study was to describe a profile of occupational therapists working in schools in three Australian states, to identify the employment and service delivery models used, to identify the client base of practitioners working in this area, and to explore how services are funded. Such a profile may establish preliminary data for future workforce planning, help prepare students for this area of practice, and assist policy makers to ensure that strategic priorities are realised.

### 4.1. Limitations and Future Research

The COVID-19 pandemic resulted in national school closures and ongoing restrictions on occupational therapists' ability to provide services in schools. It is likely that this impacted on the number of therapists willing to complete the survey and the data collected, as occupational therapists' service delivery models changed to accommodate these practice constraints.

Other limitations of the study relate to the small sample size and the recruitment of therapists from only three out of the eight Australian states and territories. The three states targeted in the study are the most populous and employ 75% of the occupational therapy workforce; however, they may not be representative of the overall school-based occupational therapy population. Compared to national figures [42], there is over representation of therapists from New South Wales and Victoria and under representation of therapists from Queensland in this study. The sample recruited within each state may also not be representative.

As there is no national registration of occupational therapists working in schools and no national workforce statistics on this population, it is likely that not all therapists working in this area were approached. A combination of sampling techniques was adopted to try to reach as many therapists as possible; however, the sampling techniques used raise issues of motivational bias due to self-selection and potential homogenous sampling as passive snowball participants volunteer other potential participants who are like themselves [43].

The survey was developed specifically for this research and this cohort of Australian occupational therapists and was pilot-tested; however, the psychometric properties of the survey are unknown which may have influenced the understanding and interpretation of questions by respondents.

Future workforce-related research would be beneficial given what is known anecdotally and supported by preliminary findings about the profile of occupational therapists working in school-based practice in Australia. The profile is incredibly varied with regard to age, experience, employment, and funding, and there is an opportunity to better understand the needs of therapists working in this complex area in relation to initial qualification, in-service education, professional competencies, and ongoing professional development requirements. This study is a beginning in identifying the change in client base as a result of inclusive education policies, as occupational therapists move from working solely with individual students to collaborating with teachers and school staff. There is scope to further identify the needs of these new client groups to understand how occupational therapy may better support them in their inclusive endeavours.

## 5. Conclusion

This study is the first to profile occupational therapists practising in mainstream primary schools in Australia. Despite its size and limitations, findings from this study begin to establish a preliminary profile of occupational therapists working in this context, the services that they provide, the types and numbers of clients, and the funding of their services. This information may be helpful for familiarising occupational therapists currently practising, or intending to practice, in the area of education and schools by elucidating the landscape of school-based practice. Understanding the work profile may support therapists to advocate using the best-practice model, the tiered model of service delivery, in their school-based practice.

Information from the study may assist service planners, policy makers, and occupational therapy educators to better understand this important and expanding area of practice to better meet the challenges of preparing and supporting the occupational therapy workforce so that clients in education contexts continue to have their occupational needs met. This data could form a basis from which a record of school-based practice throughout the nation can be made. Changes in practice can then be documented, first in relation to government policy changes, such as employment of therapists by state education departments, and second, in response to changes in priority areas of funding, for example, NDIS funding for early intervention services. As current best practice promotes the use of a tiered model of service delivery, with a focus on tier 1 universal interventions, policy makers should consider how services are funded and delivered within the education system to target inclusion in school [18]. To address future needs, ongoing, accurate SBOT workforce data could be collected and monitored to provide an up-to-date profile of school-based therapists and services. Occupational therapy workforce research can help determine whether occupational therapists exist in sufficient supply, are equitably distributed, and have the necessary knowledge and skills to practice competently and in accordance with best-practice models. Although this paper describes the workforce profile of a sample of Australian occupational therapists and is therefore unique to this context, it is likely that internationally, other countries are experiencing similar trends in workforce developments and may therefore be informed by this study. Jesus et al. [27] suggest that international coalitions are needed to support joint occupational therapy workforce developments globally, and both national and international professional associations, as the peak representative bodies for occupational therapists, play an important role in ensuring that workforce surveys occur to help with enlightened future planning of school-based therapy services.

## Figures and Tables

**Table 1:** Demographic characteristics.

	All states		New South Wales		Queensland		Victoria	
	*n*	%	*n*	%	*n*	%	*n*	%
State of employment	105	100	57	54	18	17	30	29
Gender								
Male	6	6	2	4	2	11	2	7
Female	99	94	55	96	16	89	28	93
Total^∗^	105	100	57	100	18	100	30	100
Age group in years								
Under 25	11	10.5	5	8.8	3	16.7	3	10
26-35	45	42.9	25	43.9	6	33.3	14	46.7
36-45	29	27.6	15	26.3	7	38.9	7	23.3
46-55	12	11.4	7	12.3	1	5.6	4	13.3
56-65	8	7.6	5	8.8	1	5.6	2	6.7
Total^∗^	105	100	57	100	18	100	30	100
Highest qualification earned								
Diploma	1	1	0	0	0	0	1	3.3
Bachelor's degree	74	70.5	41	71.9	14	77.8	19	63.4
Master's degree	30	28.6	16	28.1	4	22.2	10	33.3
Total^∗^	105	100	57	100	18	100	30	100
Number of years in profession								
Under 9	49	46.7	25	43.9	11	61.1	13	43.3
10-19	36	34.3	23	40.4	4	22.2	9	30
20-29	10	9.5	3	5.3	2	11.1	5	16.7
30-39	9	8.6	5	8.8	1	5.6	3	10
40-49	1	1	1	1.8	0	0	0	0
Total^∗^	105	100	57	100	18	100	30	100

^∗^Respondents did not answer every survey question; therefore, *n* = number of responses.

**Table 2:** Employment characteristics.

	All states		New South Wales		Queensland		Victoria	
	*n*	%	*n*	%	*n*	%	*n*	%
Employment hours								
Full time (>=38 hr/wk)	58	55	31	54	10	56	17	57
Part time (<38 hr/wk)	48	45	26	46	8	44	13	43
Total^∗^	106	100	57	100	18	100	30	100
Employer								
Government (health)	1	1					1	4
Government (education)	14	16	1	2	9	50	4	17
Private practice	58	64	37	79	6	33	14	58
Agency/not for profit	14	16	8	17	3	17	3	13
Other	3	3	1	2			2	8
Total^∗^	90	100	47	100	18	100	24	100

^∗^Respondents did not answer every survey question; therefore, *n* = number of responses.

**Table 3:** Service delivery characteristics.

	All states		New South Wales		Queensland		Victoria	
	*n* ^∗^	%	*n* ^∗^	%	*n* ^∗^	%	*n* ^∗^	%
School-based services provided								
Direct 1 : 1 (tier 3)	79	73.1	43	75.4	13	72.2	22	72.3
Direct small group (tier 2)	26	24.1	13	22.8	5	27.8	8	26.7
Indirect	31	28.7	12	21.1	7	38.9	11	36.7
Individual consultation	71	65.7	36	63.2	15	83.3	19	63.3
Class consultation (tier 1)	30	27.8	9	15.8	9	50	12	40
School consultation (tier 1)	24	22.2	6	10.5	7	38.9	11	36.7
School-based service provided most frequently								
Direct 1 : 1 (tier 3)	54	61	32	68.1	9	50	13	57
Direct small group (tier 2)	3	3	1	2.1			2	9
Indirect	4	5	3	6.4			1	4
Individual consultation	25	28	10	21.3	8	44	6	26
Class consultation (tier 1)	3	3	1	2.1	1	6	1	4
Location of tier 3 direct 1 : 1 school-based service								
In the student's classroom	26	33	12	28	5	38.5	8	36
Other room in the school	46	58	27	63	7	53.8	12	55
Other	7	9	4	9	1	7.7	2	9
Location of tier 2 small group school-based service								
In the student's classroom	12	46	6	46	3	60	3	37.5
Other room in the school	13	50	7	54	1	20	5	62.5
Other	1	4			1	20		
Most frequent timing of service provision								
During class teaching time	70	85.4	39	89	11	79	20	87
Outside class teaching time (e.g., recess or lunch)	1	1.2					1	4
Outside school hours prior to or after school	11	13.4	5	11	3	21	2	9
Contribution to individualised student learning/education plans (IEPs)								
Contributed to all IEPs	2	2.2			1	5.6	1	4.2
Contributed to some IEPs	44	48.9	24	51.1	4	22.2	16	66.7
Did not contribute	31	34.4	16	34	8	44.4	6	25
Unaware of students with IEPs	13	14.4	7	14.9	5	27.8	1	4.2
Grades worked with most frequently								
1st year of school (Kindy/Prep)	43	48	20	42	8	44	14	58
2nd year of school (year 1)	18	20	8	17	6	33	4	17
3rd year of school (year 2)	9	10	5	11	3	17	1	4.2
4th year of school (year 3)	7	8	4	9	1	6	2	8.3
5th year of school (year 4)	5	6	3	6			2	8.3
6th year of school (year 5)	4	4	4	9				
7th year of school (year 6)	4	4	3	6			1	4.2

^∗^Respondents did not answer every survey question; therefore, *n* = number of responses. Totals are not provided, as respondents could choose multiple options depending upon practice, e.g., direct 1 : 1 and direct small group and indirect.

**Table 4:** Client characteristics.

	All states			New South Wales			Queensland			Victoria		
	*n*	Mean	SD	*n*	Mean	SD	*n*	Mean	SD	*n*	Mean	SD
Number of teachers that the therapist is working with (*n* = 88)^∗^	88	14.1	19.6	46	12.9	10	17	14.2	11.2	24	16.8	11.4
Number of students on caseload (*n* = 89)^∗^	89	22.4	14.3	46	21.3	13.8	18	23.9	12.6	24	23.9	16.8
Known number of students on caseload with diagnosed disability (*n* = 89)^∗^	89	17.5	12	46	21.4	13.8	18	21.4	12.3	24	17.8	13.7
Known number of students receiving adjustments per NCCD (*n* = 85)^∗^	85	11.1	11	44	9.2	10.3	18	14.3	12.9	22	12.6	10.7
Number of students on caseload receiving school-based services (*n* = 88)^∗^	88	13.7	11.6	46	13.2	11.1	18	17.1	13.9	23	12.1	10.7

^∗^Respondents did not answer every survey question; therefore, *n* = number of responses.

**Table 5:** Funding of therapy services.

	All states	New South Wales	Queensland	Victoria
	%	%	%	%
NDIS funding	76	85	47	78
Private funding	19	27	24	19
Department of Education funding	20	7	56	16
Catholic education/independent system funding	8	7	19	16
Individual school funding	11	7	24	20

## Data Availability

The data is hosted by the University of Sydney as it forms part of data collected for a doctoral thesis.
